# High emission reduction performance of a novel organic-inorganic composite filters containing sepiolite mineral nanofibers

**DOI:** 10.1038/srep43218

**Published:** 2017-03-02

**Authors:** Fei Wang, Hui Zhang, Jinsheng Liang, Qingguo Tang, Yanxia Li, Zengyao Shang

**Affiliations:** 1 Institute of Power Source & Ecomaterials Science, Hebei University of Technology, 300130 Tianjin, China; 2 Key Laboratory of Special Functional Materials for Ecological Environment and Information, Hebei University of Technology, Ministry of Education, Tianjin 300130, China

## Abstract

In this work, a new organic-inorganic composite filter was prepared. The thickness, pore size, air permeability, bursting strength and microstructure were characterized systematically, proving that coatings had regulatory effect on filters physical properties. Benefitting from the distinct coatings containing 5% sepiolite nanofibers after five times dilution, the physical properties of corresponding air filter exhibits the most favorable performance and meet the standard of air filter. When used as fuel filter, it satisfies the fuel filter standard and achieves the best performance after six times dilution. The contrast test on engine emission was taken based on auto filters coated with/without as prepared nanofibers. An obvious decrease in the emission of carbon monoxide (CO), hydrocarbons (HC) and nitrogen oxide (NO_x_) can be observed after installation of composite filter on vehicles. Under the high idle condition, gasoline engine emission decreased by 8.13%, 11.35% and 44.91% for CO, HC and NO_x_, respectively. When tested in the low idle condition, engine emission reduced by 0.43%, 1.14% and 85.67% for CO, HC and NO_x_, respectively. The diesel engine emissions of CO, NO_x_ and total amount of HC and NO_x_ decreased by 32.26%, 3.28% and 4.66%, respectively. The results illustrate the composite installation exhibits satisfactory emission reduction effect.

Resources and environment have been global ticklers that beset the sustainable development of human society. As the demands progress, the mobile equipments are impelled to the light and high efficiency. In recent years, with atmosphere pollution being more serious, e.g. smog, in large and medium sized cities of China, there are higher requirements on reduction of automobile emission. Especially in aspect of air pollution, according to the standard of EURO V, the particulate emissions of diesel vehicles are expected to decline 80% in comparison with the present. On the other side, the nitrogen oxides emissions of diesel engine shall decrease 68%, according to the standard of EURO VI. The development of high efficiency emission reduction technology has been explored worldwide in response to the challenge of environment pollution^1^,^2^.

It is known that the harmful substances in the automobile exhaust mainly consist of hydrocarbons, carbon monoxide, nitrogen oxides, sulfur compounds, smoke, etc. Among them, carbon monoxide, hydrocarbons and nitrogen oxides are the main air pollutants^3^,^4^,^5^,^6^,^7^,^8^. The control methods to reduce vehicle pollutant emission include internal engine purification, external engine purification and fuel oil improvement. Among them, the internal and external purification methods have been the worldwide research hotspot in the technology of emission control^9^,^10^,^11^,^12^,^13^,^14^,^15^,^16^,^17^. As one critical component of external purification system, auto filters becoming increasingly significant in separation of harmful impurities and moisture from either the air or fuel. To achieve effective reduction on the harmful gases of automobile emissions while claiming no increase in the burden of technology, it is of great significance to prevent the spread of pollution from the source through developing filtering materials of high purifying efficiency.

Sepiolite is a type of fibrous silicate clay mineral rich of magnesium with a unit cell formula of Si_12_O_30_Mg_8_(OH)_4_(H_2_O)_4_·8H_2_O and fine microporous channels of dimensions of 0.37 nm × 1.06 nm running parallel to the direction of fibers^18^,^19^,^20^,^21^. Benefitting from its large reservation, low cost, high efficiency and reusable attribute, sepiolite has been intensively studied in recent years^22^,^23^,^24^,^25^,^26^,^27^,^28^. Although the defibering treatment of sepiolite is a simple and cheap way to obtain mineral nanofibers for practical use, it has been less investigated except for a few exploratory researches which mostly involve destruction of its fibrous micro-morphology by ball milling and the use of expensive and sensitive agents^29^,^30^.

In our previous research, we have prepared sepiolite nanofibers using high speed airflow technique^31^,^32^. In this work, a new kind of multifunctional organic-inorganic composite filter material was prepared. The influence of composite coating and addition amount of sepiolite nanofibers on the filters properties have been explored, and the optimized ratio of coating materials and nanofibers was obtained. Besides, in order to study on the discharge performance of the composite filters, the contrast tests of the exhaust gas emission from gasoline engine and diesel engine were also conducted.

## Methods

### Materials

The wood pulp was purchased from Henan Huining Trade Co., Ltd. The synthetic fiber was supplied by Zibo Ruibao Materials Co., Ltd. The sepiolite as one of natural mineral materials was purchased from Henan province of China, and the high performance sepiolite mineral nanofibers (SMN) preparation has been studied previously^31^,^32^. The silane coupling agent (KH560) was supplied by Nanjing Shuguang Chemical Group Co., Ltd. The air and fuel filter paper were purchased from Hangzhou Xinxing Co., Ltd and used as the substrate. All of the compounding ingredients were commercially available in chemical store.

### Preparation

**Figure 1:**
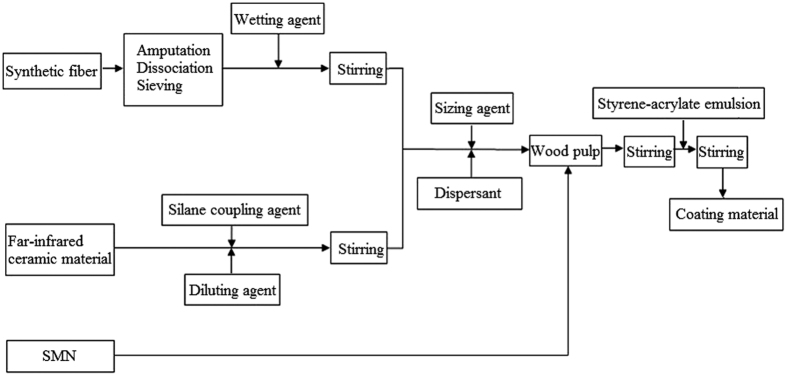
Preparation process of composite coating material.

The preparation process of composite coating material was shown in Fig. 1, and the coating preparation process was shown as follows. The coating material was covered evenly on the outflow surface of substrate and dried to obtain the composite filter samples. During the preparation process, the coatings must be coated evenly on the outflow surface of substrate with so much attention as the range of 30~60 min after the coating materials were ready.

### Measurement

An Olympus STM6 metallographic microscope and a Hitachi S-4800 scanning electron microscope operated at 25.0 kV and 30 μA were used to study sample morphological features. After cutting the composite filter material into the size of 100*100 mm, quantitative tests of filter paper were carried out employing a precision electronic balance (Sartorius Company, German) to weigh the samples. In our study, paper thickness tester was performed via the measurements of the samples according to the China Standard Test Methods (QB/T 1055091, Paper and Cardboard Thickness Measuring Instrument). At least 5 samples with the dimensions of 100*100 mm were cut from each test piece, placed between the measuring head and the anvil and given with certain hammer pressure. The samples thickness was delivered to the gear mechanism of watch via the displacement of the measuring rod, where it let the watch hands rotate an angle to obtain the thickness. The thickness was measured at least 3 times at each position and the average value was recorded as the result. Air permeability, a common detection index of air filtration materials, referred to the gas volume through unit area of materials under unit time and pressure, and reflected the gas permeation ability of filters. Air permeability test was carried out according to the China Standard Test Methods (GB/T5453-1997, determination of fabric permeability). There was no direct conversion relationship between the pore size and filtration precision of filters. However, the filtration precision was reflected indirectly via the aperture test which referred to the principle of capillary flow according to the China Standard Test Methods (QC/T794-2007). Bursting strength was the maximum pressure which was endured evenly increasing in the unit area and denoted by liquid pressure or gas pressure. The tests were performed using electronic paper bursting tester (ZDNP-1) according to the China Standard Test Methods (QB/T Bursting Strength of Paper and Paperboard). According to measurement method of fuel saving technology for automobiles (GB/T 14951-2007), the pollutant emissions of engines were detected. On the other side, the effects on the emission reduction and environmental protection of air filter and diesel filter were also tested, according to the emissions of pollutants from light-duty vehicles limit values and measurement methods in China III, IV stage (GB/T 18352.3-2005).

## Results and Discussion

**Figure 2:**
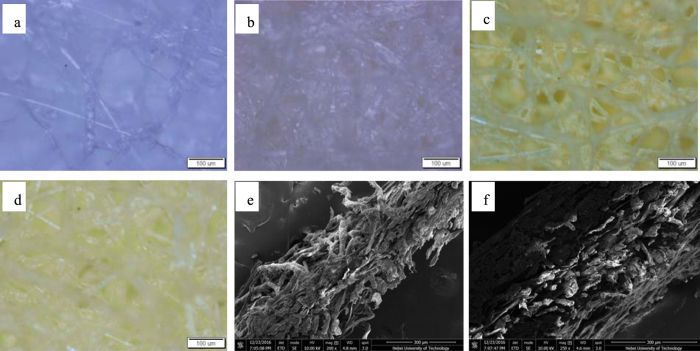
Microstructure of different samples. (**a**) Air filter paper; (**b**) Fuel filter paper; (**c**) Air composite filter; (**d**) Fuel composite filter; (**e**) Sectional microstructure of air composite filter; (**f**) Sectional microstructure of fuel composite filter.

The contrast observation was carried out with the resultant samples, the air composite filter material with 5 times dilution of the coating material and the fuel oil filter material with 6 times dilution of the coating material. In this study, the air filter paper and the fuel oil filter paper were chosen as the substrate, and the microscopic observation was conducted on the outflow surface of raw material samples. The distribution changes of fibers and pore sizes in two types of composite filters were presented in Fig. 2a and b. After the coating treatment, the contrast observations on the air and fuel filter paper were monitored under the same field of view and the same magnification, as shown in Fig. 2c and d. In addition, the sectional microstructure of the air and fuel composite filter was indicated in Fig. 2e and f, respectively.

As observed in Fig. 2a, the wood fibers in the air substrate filter paper are coarse, mixed and random. In addition, the pores of the fiber are large and uneven, the pore size is up to approximately 300 μm, and the porosity is about more than 70%. Figure 2b shows fiber structure of fuel oil filter paper at room temperature, which can be clearly seen the fuel filter paper is composed of the staggered reticular wood fiber. Compared with the air filter paper (Fig. 2a), the fibers of fuel filter paper (Fig. 2b) show the close distribution, and the fiber packing density is approximately more than 60%. Figure 2c shows fiber distribution of the air flow surface composite filter. Compared with the air substrate filter paper (Fig. 2a), the composite filter fibers (Fig. 2c) are relatively dense, and some tiny fibers can be monitored among the wood fibers under the high magnification observation. In addition, the pore distribution is more evenly, the fiber packing density obviously increases to more than 50%, and the pore size reduces to 40–200 μm. Figure 2d shows fuel oil fiber distribution of the air flow surface composite filter. Compared with the fuel oil substrate filter paper (Fig. 2b), the fibers with different thickness have the obviously staggered reticular structure, and the pore distribution is more evenly. This behavior is attributed to that the coating material is mainly composed of wood pulp fibers and synthetic fibers with different length versus diameter ratio, and the fiber hybrid increases the fiber density on the composite material surface, forming a more complex three-dimensional network structure. As could be seen from Fig. 2e and f, there is no obvious gap between the fibers of composite filters and substrate. Direct congruity’s method as the common composite technology in the organic-inorganic composite filter section takes place unevenly in composite layer and has a serious influence on the filtration separation process, introducing the error to the performance test and leading to questionable conclusions. Using our method, the loose hydrogen bonds between the interlayer of composite materials are formed due to the dipolar water molecules and fibers after the adoption of the coating treatment. The hydrogen bonding force connects the fibers between the layers, improving the strength between the layers.

The as-prepared coating material, possessing the properties of high viscosity and concentration, was not suitable for the direct preparation of the coating. Therefore, the coating material needed to be diluted with a certain amount of water in order to ensure the performance of composite filters. In our study, the as-prepared coating material was denoted as the standard solution with 100% concentration and diluted according to solution/water ratio of 1:1,1:2,1:3,1:4,1:5 and 1:6, corresponding to dilution times of 2, 3, 4, 5, 6, and 7, respectively. The diluted coating materials were coated and dried on the substrate, and tested on the physical performance.

**Figure 3:**
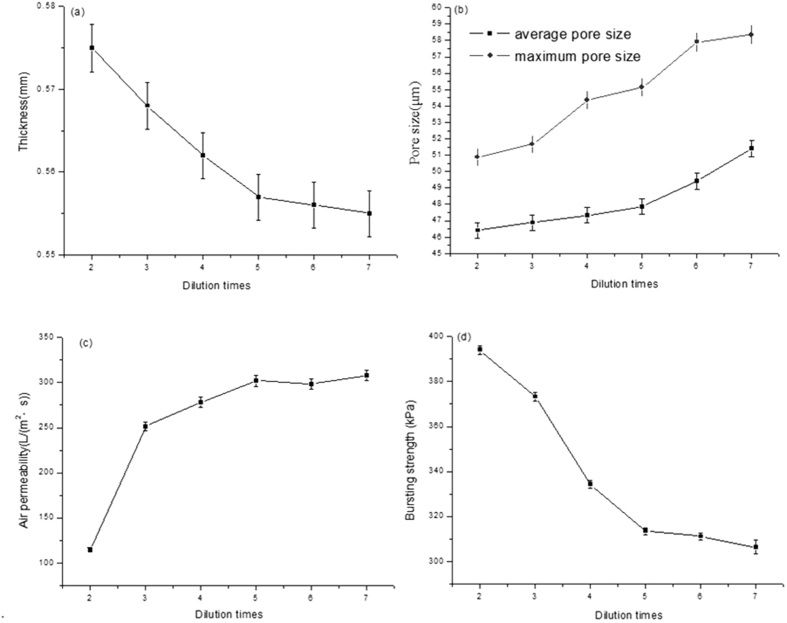
The effect of the coating materials dilution times on the physical performance of air composite filters. (**a**) Effect on the thickness; (**b**) effect on the pore size; (**c**) effect on the air permeability; (**d**) effect on the bursting strength.

The effect of coating materials dilution times on the physical performance of air composite filters is shown in Fig. 3.

In this method, the material construction mainly depends on the bonding strength between layers, ensuring the tight binding between layers without adopting any inter layer pressure, which yields identical material density. Therefore, the amount of the composite coating after drying can be directly denoted as the change of composite materials thickness. As presented in Fig. 3a, the curve of the composite filter material thickness is smooth with the amount variation of coating material and has no phenomenon of sudden increase and decrease, illustrating that there is no obvious gap between the composite filter coating layer and base layer and verifying the structural design theory indirectly. The downward tendency in the total composite filter material thickness is observed with increasing coating material dilution. With increasing the dilution times to 5 and decreasing the thickness to 0.55 mm, the tendency in the thickness change is stable. This is possibly ascribed to the following reasons: the coating materials exhibit high concentration and viscosity, the fine fibers distribute unevenly, and the fibers are easily aggregated into large fibers. It is difficult to enter into the basic fiber surface voids combining with the substrate during the coating process. With increasing dilution times, the fluid viscosity decreases, the fine fiber can be distributed evenly in the fluid, the probability of contact with the base layer increases during the fluid flow process, and the amount of the pores entering on the substrate surface increases. With the reduction to a certain extent, tiny fibers are more likely to enter into the pores of the substrate surface with the fluid, as the thick fibers are boarded on the surface. With increasing the dilution times, the low amount of coating layer fiber has no significant influence on the total thickness, and finally tends to be stable.

As is clearly observed in Fig. 3b, with increasing the coating material dilution times, the amount of coating decreases and the tendency in the average pore size and maximum pore size of composite filter material is upward. Furthermore, with increasing the dilution times, the maximum and average pore size is 54.33 μm and 47.31 μm, respectively, and the growth trends of maximum pore size and average pore size are large. It can be put down that the decreasing amount of fiber in coating layer leads to the less complexity of fiber structure and the larger pore size in layer. It also can be seen from Fig. 3b that the difference between the maximum and average pore size increases with the decrease of the coating amount, illustrating that pore size distribution of filter material is more uneven with decreasing the coating amount. This is possibly attributed to the decrease amount of fiber in coating layer, the degree of interwoven at the reduced fiber and the much weaker regulating action to the pore size. Figure 3c shows with increasing the coating material dilution times, the rising tendency in air permeability of composite filter is obvious and the maximum rising trend takes place at the dilution times of 2 and 3. This phenomenon is ascribed to that the coating amount of composite filter is large and the fibers distribution is uneven with decreasing the coating dilution times, resulting in pore blockage of composite filter and air permeability decreasing obviously. With increasing the dilution times more than 5, the fibers of coating layer and addition materials reduce and the dispersion property is more even, which makes tiny fibers and layer fibers form a three-dimensional network structure more easily.

The effect of dilution times of coating material on bursting strength is shown in Fig. 3d. The coating layer contains a certain amount of synthetic fibers, exhibiting the characteristics of small density, smooth surface, straight fiber and good elasticity, which can improve the permeability and strength of the fluid. Figure 3d shows that the bursting strength of composite filter material shows a downward trend along with the increase of the dilution times. The phenomenon is ascribed to that the large amount of composite fiber mainly increases the strength of the role in high concentration of coating materials and the strong winding effect of fibers results in the high bursting strength of composite filter in high concentration. With increasing dilution times and decreasing coating amount, the fibers interaction also weakens and the mechanical properties of the composite filter decrease significantly. It is also indicated that the reduction of bursting strength achieves 60KPa with increasing the dilution times from 2 to 5. As increasing the dilution times from 5 to 7, the reduction is only 7kPa and the change tends to be flat. This is attributed to that the synthetic fibers amount in coating layer decreases to a certain degree, which no longer plays an obvious role in the strengthen function and mainly relies on the other fibers of coating layer interweaving so as to increase bursting strength.

Together the effect of different dilution times and coating amount on physical properties of composite materials above, we can see that the physical properties of the coating material could be adjusted through adding a certain amount of composite materials in a certain range. According to the air filter production requirements, each air filter index must be in line with the relevant standards, as shown in Table 1. In combination with the test values of various performance indexes, the 5 times dilution of the coating material is selected as the optimal ratio for the air composite filter. At this point, physical performance indexes of the composite air filter are that thickness, maximum and average pore sizes, permeability and bursting strength is 0.56 mm, 54.33 μm, 47.31 μm, 302 L/(m^2^·s) and 313KPa, respectively.

**Figure 4:**
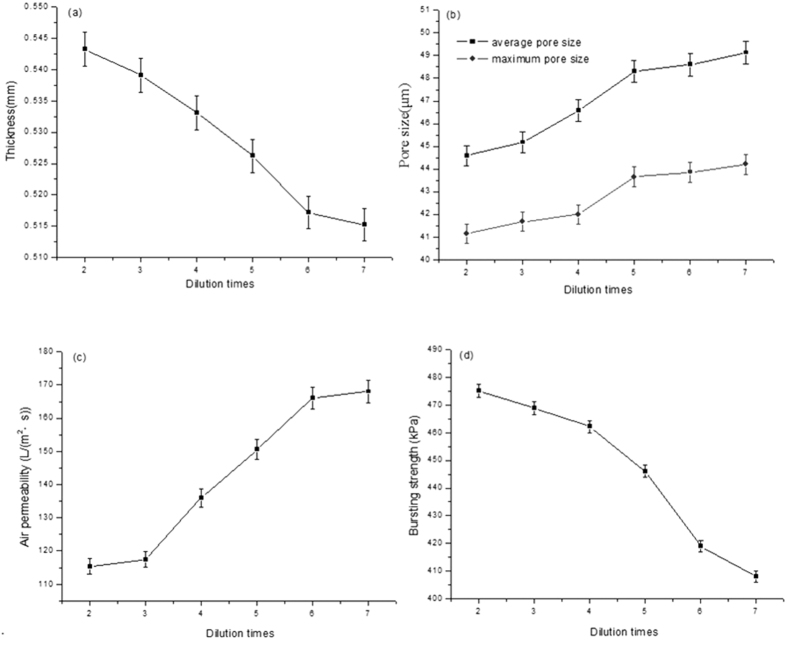
The effect of the coating materials dilution times on the physical performance of fuel composite filters. (**a**) Effect on the thickness; (**b**) effect on the pore size; (**c**) effect on the air permeability; (**d**) effect on the bursting strength.

The effect of the coatings dilution times on the physical performance of fuel composite filters is shown in Fig. 4. According to the above analysis of the effect of composite air filter’s coating material dilution times on the thickness of the filter, theoretically at the same proportion, the fuel composite filter thickness has the same variation tendency as the air composite filter thickness. As shown in Fig. 4a, the composite fuel oil filter thickness has the same trend as the overall air composite filter. However, as diluting the coating material 6 times, the composite fuel filter thickness appears even. The possible factor of phenomenon is mainly due to that the pore size distribution of fuel substrate is denser than that of the air substrate. With diluting the coating material 5 times, the coating amount could not meet the requirement that the slender fibers are completely contacted with the substrate surface. Another is ascribed to that the difference is caused via the substrate flatness or the errors during the measurement process. As can be shown in Fig. 4b, with increasing the dilution degree and reducing the coating amount, the maximum and average pore size of composite fuel oil filter shows the increasing trend and the difference between maximum and average pore size gradually increases from 3.44 to 4.92 μm, indicating the pore size distribution of burning oil composite filter becomes gradually uneven with decreasing the coating thickness. Compared with the trends of air filter pore size, two kinds of curves exhibit the similar tendencies. The difference is that composite fuel oil filter tends to be stable with diluting coating material 6 times. This is attributed to that the outflow fibers of fuel oil substrate filter arrange compactly, the surface aperture is smaller than air substrate, and the extent of coating is required differently from that of air substrate filter. It is clearly seen from Fig. 4c that the permeability of multilayer composite filters is less than that of each layer, depending on the smaller size layer. As increasing the coating materials dilution times, the coating amount of composite filters decreases, the coating layers complexity reduces and the air permeability increases, resulting in the overall air permeability of composite filters increasing. When the air permeability of coating layer reduces to a certain extent, the air permeability of composite filters tends to be stable, close to the air permeability of original substrate filter paper. As shown in Fig. 4c, the air permeability of fuel oil composite filter increases with the high dilution times of coating materials. The augment of air permeability is the most obvious within the range of 2–6 times. When the dilution times increases to 6, the air permeability attains to 166 L/(m^2^·s). However, when the dilution times continue increasing, the curve of air permeability variation levels out. As shown in Fig. 4d, with increasing the coating materials dilution times, the bursting strength of composite filter decreases. In other words, the bursting strength of composite filter reduces with decreasing the coating amount, indicating that the coating material exhibits the enhancement of the filter strength function. It also can be seen that the bursting strength reduces distinctly in the dilution range of 2–6 times; when the dilution times is larger than 6, the bursting strength change of composite filter begins to be stable. With the coating amount variation, the bursting strength of fuel oil composite filter is broadly consistent with that of air composite filter, which validates the theory that the coating material has the effect on the bursting strength. The integrated experimental results of thickness, pore size, air permeability and bursting strength indicate that the bursting strength of fuel oil composite filter gets evenly when the coating material dilution times is higher than 6, depending on the interaction with the coating material and fuel oil substrate.

The change trend of different dilution times and coating material amount effect on the physical properties of oil composite filter states that the physical properties of composite filter can be adjusted within a certain range via adding a certain amount of coating material. According to fuel oil filter production requirements, each physical properties index of fuel oil filter must meet the related standard of fuel oil filter as shown in Table 2. In combination with the standard value of each performance index, the composite fuel oil filter exhibits the optimal parameter as diluting coating material 6 times.

**Figure 5:**
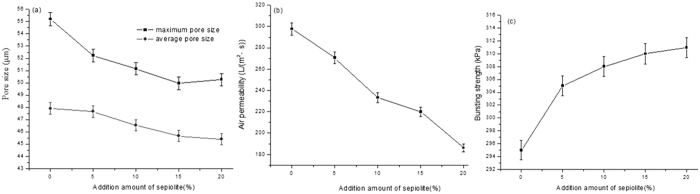
The effect of the SMN addition amount on the physical performance of air composite filters. (**a**) Effect on the pore size; (**b**) effect on the air permeability; (**c**) effect on the bursting strength.

The effect of the addition amount of SMN on the physical performance of air composite filters is shown in Fig. 5. As shown in Fig. 5a, the maximum and average pore sizes of composite air filter material show a downward trend with adding a certain amount of SMN. The decrease amplitude of maximum pore size is slightly larger than that of average pore size, and the difference between maximum and average pore sizes shows a trend of decreasing at first and then increasing slightly. When the addition amount of SMN attains to 5%, the difference value achieves the minimum value of 4.23 μm at first and then increases to 4.87 μm, which indicates the composite filter aperture varies evenly, increasing firstly and then decreasing. When the addition amount range is 0–10%, the downward trend of maximum pore size and average pore size is large. When the addition amount is higher than 15%, it tends to be even. This phenomenon is possibly attributed to that sepiolite is a kind of tiny fiber, and the SMN form the complex network structure with fiber carrying in the filters when increasing its content. When the addition amount is large to an extent, the uneven dispersion of SMN results in the aggregation and SMN does not work on the large pores adjustment on the fiber surface any more.

Figure 5b shows that with increasing the SMN addition amount, the air permeability curve of air composite filter decreases obviously. This phenomenon is ascribed to that when the SMN addition is too high, the SMN are mixed with other fibers, affecting the air permeability of composite filter material and leading to pore clogging and shorter life when the situation is serious. According to the air filters production requirements, the air permeability standard of air filter in production should be not less than 260 L/(m^2^·s). The figures above present when the addition amount is more than 10%, the air permeability of the filter material decreases below to 223.3 L/(m^2^·s), which does not meet the standard. Figure 5c shows that with increasing the SMN addition amount, the bursting strength of air composite filter material increases. When increasing addition amount in the range of 0–10%, the bursting strength increases the most obviously, however, when the addition amount is higher than 10%, it tends to be stable. This phenomenon may be attributed to that with the little addition amount, the SMN is easy to disperse and distribute evenly among the various kinds of fibers, indicating that the interaction improves between the fibers and the strength of filter material enhances effectively. When the addition amount is large to a certain amount, the SMN distribution is uneven and causes the aggregation easily, which does not make the SMN fully utilized.

As discussed above, the SMN addition amount can adjust the composite filter pore size and increase bursting strength within a certain range, but the air permeability decreases with increasing the SMN addition amount. According to the air filter production requirements, the air filter indexes must comply with the relevant standards, as shown in Table 1. As the trend analysis of various performance indicators, 5% is chosen as the optimal SMN addition dosage. The optimal air composite filter ratio is to dilute coating material 5 times, including 5% of SMN.

**Figure 6:**
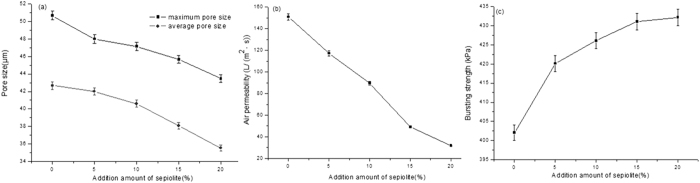
The effect of the SMN addition amount on the physical performance of fuel composite filters. (**a**) Effect on the pore size; (**b**) effect on the air permeability; (**c**) effect on the bursting strength.

The effect of the addition amount of SMN on the physical performance of fuel composite filters is shown in Fig. 6.

As shown in Fig. 6a, it can be seen that with the variation of SMN addition amount, the pore size of fuel filter material has the same tendency as that of air filter material. With increasing the SMN addition amount, the changes in the maximum and average pore sizes show a downward trend. The difference between maximum and average pore size changes from large to small, and then increases slightly. When the addition amount turns to 5%, the difference reaches to the minimum 5.95 μm, which demonstrates the uniformity variation of composite filter material’s pore size changes from small to large, then slightly decreases and reaches the maximum at 5%. It can be also seen from Fig. 6a that the maximum and minimum pore size difference of composite fuel oil filter material reaches the minimum at 5% of SMN amount. This is probably attributed to that the fuel oil substrate surface pore is compact, hence the tiny fibers intake capacity is little. When it exceeds a certain amount, the fibers may generate the clusters, which is no longer effective in the pore size adjustment.

From Fig. 6b, with increasing the SMN addition amount, the air permeability of composite filter material has obvious downward trend. When the SMN addition amount are 15% and 20%, the air permeability drops to 38 L/(m^2^·s) and 27 L/(m^2^·s) respectively. This is ascribed to that the dense fibers of fuel oil substrate show smaller intake capacity to sepiolite. When the addition amount reaches to a certain amount, the SMN forms the blockage to the filter material pores. Fuel filter standard states that the air permeability is not less than 80 L/(m^2^·s), as shown in Table 2. Hence, when the SMN addition amount exceeds 10%, the air permeability of composite fuel oil filter material can not meet the standard. The air permeability is an important filter material index. The small air permeability causes the lack of oxygen in the combustion chamber, the increase of air-fuel ratio, the rise of fuel consumption and the increase of pollutant emission. The optimal selection should be under the comprehensive consideration on other performance indicators and standards. According to the comparison with the curves of air filter material and SMN, it is found that two charts have the similar trend, indicating that the basic principle of the SMN effect on the performance of filter material is similar. As shown in Fig. 6c, it can be seen that with increasing the SMN addition amount, the bursting strength of composite filter material presents obviously rising trend, from 402 to 433kPa, illustrating that the SMN addition has a certain effect on the strength of composite filters, and the growth trend rises from large to small. Comparisons with the curves of air filter material and SMN show that two charts have the similar tendency, which verifies the theoretical analysis study on the bursting strength variation of composite air filter material.

As the analysis above, it is shown that the trends on performance indexes of the composite fuel oil filter material with the addition amount variation of SMN and composite air filter material are alike, which validates the analysis of the SMN dosage effect on the composite filter material and states that the effect of SMN dosage on composite filter material is consistent with the fact. According to the production requirements, performance indexes of fuel oil filter material have to meet the criteria as shown in Table 2. Considering these indexes, 5% is selected as the optimal dosage. Hence, when the coating material dilution times are 6 and the SMN addition amount in the coating material is 5%, the performance indexes of composite fuel oil filter material achieve the best.

For the sake of the detection on the effect of organic-inorganic composite filter material on the emission of gasoline vehicles, organic-inorganic composite filters are prepared to a certain type of air filter and fuel filter, which are record as experiment group of air filter and experimental group of gasoline filter. The untreated substrate filter paper is selected to fabricate the control group of air filter and gasoline filter with the corresponding type. According to GB/T 14951-2007, the pollutant emissions of engines are detected, and the contrast test results on the pollutant emission of gasoline vehicles are shown in Table 3.

Table 3 shows that under the high idle condition, the contrast tests between the harmful gas emissions of the experimental group engine and the control one indicate that the emission of harmful gases CO, HC and NOx in experimental group decreases. The reduction ratios of CO, HC and NO_x_ are 8.13%, 11.35% and 44.91%, respectively. Under the low idle condition, the reduction rate of CO, HC and NO_x_ is 0.43%, 1.14% and 85.67%, respectively.

The design on the physical and chemical properties improvement of vehicle fuel and activating fuel is integrated into the composite filter material. According to the combustion principle of internal combustion engine, it has been stated that the surface tension of the fuel oil decreases and the evaporation property improves, which is benefits to the complete atomization of the fuel oil and promote the oxidation reaction of the fuel oil molecule and oxygen, so as to achieve the goal of emission reduction of HC, CO and NOx in tail gas. Far infrared functional material has the function of releasing far infrared ray. After the far infrared ray is absorbed by fuel oil, the fuel surface tension reduces and the atomization improves, so that it can promote the fuel complete combustion.

A method for judging the combustion efficiency is by means of the combustion product definition. This method depending on the instrument measurement, which is not related to the fuel application, has been worldwide adopted. CO and HC, the form of unburned carbon element, which content can directly reflect the complete extent of the fuel combustion, is an important symbol of the combustion efficiency. By means of gas analysis method, the contents of HC, CO, CO_2_ and NO_x_ in combustion products from the combustion chamber are measured, and the combustion efficiency is determined. The expression of combustion efficiency is:


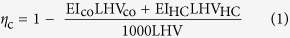


In formula: EI is the mass of the pollutants produced by each kilogram of fuel, g/kg. LHV represents low calorific value of combustion, MJ/Kg.

The unburned hydrocarbons (HC) in tail gas emissions are the important symbol to reflect the complete extent of the fuel combustion. As it can be seen from the test data, the content of HC in the engine combustion products of experimental group is less than that in the control group. It shows that the fuel combustion complete extent in the experimental group is higher than that in the contrast group, which explains the combustion efficiency is higher. The CO in the tail gas is a form of unburned carbon element in fuel. The content of CO reflects the complete degree of the fuel combustion and affects the combustion efficiency. The reduction amounts of CO and HC are put into the formula(1), so the combustion efficiency of the experimental group is expressed as:


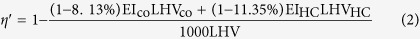


It can be seen from that formula, when the HC and CO harmful gases reduce, the combustion efficiency of the experimental group is higher than that of the control group. The gasoline combustion efficiency increases, the harmful gas emission reduces, and the effect of emission reduction is achieved. It shows that the structure and properties of composite materials match the models.

In order to detect the effect of organic-inorganic composite filter material on the pollutant emission of diesel vehicles, organic-inorganic composite filters are prepared to a certain type of air filter and fuel filter, which are record as experiment group of air filter and experimental group of gasoline filter. The untreated substrate filter paper is selected to fabricate the control group of air filter and gasoline filter with the corresponding type. According to GB/T 18352.3-2005, the effects on the emission reduction and environmental protection of air filter and diesel filter are detected, and the contrast test results on the pollutant emission of gasoline vehicles are shown in Table 4.

As shown in Table 4, under the same operation condition, the emission of harmful gases CO, HC and NO_x_ in experimental group decreases in comparison with that of the control group. The reduction rate of CO, HC, the sum of HC and NO_x_ is 32.26%, 3.28% and 4.66% respectively, which achieves the effect of emission reduction. CO and HC are two kinds of unburned carbon in the fuel, which content directly reflects the degree of fuel combustion. The values of CO and HC are put into the formula (1), so the combustion efficiency is expressed as:


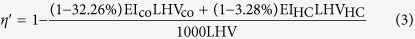


It can be seen from the formula above that the combustion efficiency is improved obviously, when the emission amounts of CO and HC are decreased. The main reason could be that when the diesel fuel flows through with far infrared radiation ceramics filter, physicochemical properties of diesel are improved and the extent of diesel atomization is enhanced. Accordingly, the diesel is burned completely in the engine, and the emission could be reduced effectively. The experiment results show that the structure design of composite materials is reasonable and exhibits good performance.

## Conclusions

In our study, a novel type of multi-functional organic-inorganic composite filter material for emission reduction was prepared, and the structure and performance were also studied systematically. The results show that coating amount exhibit positive influence on physical performance of composite filter material. When the coating material was diluted to 5 times and the SMN addition amount was 5%, the composite air filter achieves the best physical performance. Meanwhile, all the indexes of as prepared composite meet the standard of air filter material with the material thickness of 0.554 μm, the maximum pore size of 52.14 μm, the average pore size of 47.63 μm, the air permeability of 270.8 L/(m^2^·s), and the bursting strength is 305kPa. When the coating material was diluted by 6 times and the SMN addition amount maintained 5%, the physical performance of fuel filter material achieves the best. Meanwhile, the filter material thickness is 0.52 μm, the maximum pore size is 45.38 μm, the average pore size is 416 μm, the air permeability is 128.37 L/(m^2^·s) and the bursting strength is 416kPa, satisfying all the standard of fuel filter material. In the tests of emission reduction performance on gasoline and diesel vehicles, obvious decrease in the emission amount of CO, HC and NO_x_ has been detected after installation of the composite filter. In particular, the gasoline engine emission of CO, HC and NO_x_ decrease by 8.13%, 11.35% and 44.91% respectively under the high idle condition. Under the low idle condition, the emission of CO, HC and NO_x_ reduce by 0.43%, 1.14% and 85.67%, respectively. On the other hand, the diesel engine emissions of CO, HC and the total amount of HC and NO_x_ decrease by 32.26%, 3.28% and 4.66% respectively. The results demonstrate that the good effect of emission reduction can be achieved by utilizing auto filters fabricated from our nanofibers with composite coating.

## Additional Information

**How to cite this article:** Wang, F. *et al*. High emission reduction performance of a novel organic-inorganic composite filters containing sepiolite mineral nanofibers. *Sci. Rep.* **7**, 43218; doi: 10.1038/srep43218 (2017).

**Publisher's note:** Springer Nature remains neutral with regard to jurisdictional claims in published maps and institutional affiliations.

## Figures and Tables

**Table 1:** The standard of air filter.

Index name	Unit	Standard value
Quantity	g/m^2^	130 ± 10
Thickness	mm	0.4–0.6
Permeability (Δ = 200 Pa)	L/(m^2^·s)	≥260
Bursting strength	kPa	≥250
Maximum pore size	μm	≤80
Average pore size	μm	≤70

**Table 2:** Fuel filter standard.

Index name	Unit	Standard value
Quantity	g/m^2^	135 ± 10
Thickness	mm	0.4～0.7
Permeability (Δ = 200 Pa)	L/(m^2^·s)	≥80
Bursting strength	kPa	≥300
Maximum pore size	μm	≤65
Average pore size	μm	≤50

**Table 3:** Testing data of pollutant.

Test item	Control group		Experimental group	
	High idle	Low idle	High idle	Low idle
CO(%)	2.09	2.34	1.92	2.33
HC(10^−6^)	141	175	125	173
NO(10^−6^)	344	157	189.5	22.5

**Table 4:** Testing data of pollutant.

Test sample	CO (g/km)	HC (g/km)	HC + NO_x_ (g/km)
Control group	0.031	0.549	0.579
Experimental group	0.021	0.531	0.552
